# Research hotspots and development trends of *Brucellar* spondylitis in the past 30 years: a bibliometric analysis

**DOI:** 10.3389/fmicb.2025.1541792

**Published:** 2025-02-19

**Authors:** Jinyu Wu, Hongxia Yuan, Hongchao Chen

**Affiliations:** ^1^The First Affiliated Hospital of Jinzhou Medical University, Jinzhou, Liaoning, China; ^2^Liaoyang Infectious Disease Hospital, Liaoyang, Liaoning, China

**Keywords:** *Brucella*, *Brucellar* spondylitis, infectious disease, bibliometric analysis, CiteSpace, VOSviewer

## Abstract

**Objective:**

With the growing emphasis on global health issues, *Brucellar* spondylitis, a serious complication of brucellosis, has received increasing attention from researchers. This study employed bibliometric analysis to visually illustrate the scientific advancements and research trends in the field of *Brucellar* spondylitis research, providing a scientific basis for public health prevention and control strategies.

**Method:**

The data were sourced from the Web of Science Core Collection database, covering the period from January 1, 1984, to December 31, 2023. Network analyses of countries, institutions, authors, and keyword collaborations, as well as co-citation analyses of journals and references, were conducted using CiteSpace software. VOSviewer software was used to analyze the co-occurrence and hotspots of keywords.

**Result:**

A total of 246 relevant documents were retrieved, comprising 221 articles and 25 review articles. From 1984 to 2023, the number of research publications exhibited a generally fluctuating upward trend. Turkey and China emerged as the primary contributing countries in this field, with Xinjiang Medical University identified as the most productive research institution. Additionally, Juan D. Colmenero is recognized as the author with the highest number of published articles and citations. “Clinical Infectious Diseases” is regarded as the most influential journal in this domain. Among the 404 keywords analyzed by CiteSpace, the top 10 high-frequency keywords included spondylitis, complication, melitensis, osteoarticular complication, diagnosis, infection, clinical characteristics, epidural abscess, arthritis, and feature. A total of 14 clusters were formed. In the burst analysis of the top 15 keywords, “instrumentation” exhibited the highest burst intensity, while “arthritis” demonstrated the longest burst duration. Emerging keywords include “experience,” “ankylosing spondylitis,” “China,” and “instrumentation.”

**Conclusion:**

This study is the first bibliometric analysis in the field of *Brucellar* spondylitis, which revealed that the research hotspots in this field included the clinical characteristics of the disease, the management of complications, and treatment strategies. The development trend may involve enhancements in early diagnostic methods and advancements in surgical instruments. This study serves as a valuable reference for future research directions.

## Introduction

*Brucellar* spondylitis is one of the most common complications associated with brucellosis, a natural zoonotic infection transmitted from animals to humans due to brucellosis infection (Fang et al., 2022). The disease primarily spreads through contact with infected animals or animal products, as well as through the consumption of unpasteurized dairy products (Głowacka et al., 2018). Recent years have seen a gradual increase in the incidence of this disease (Liu et al., 2023). Brucellosis is widely distributed globally, with epidemics occurring in more than 170 countries and regions. Approximately 500,000 cases of human brucellosis are reported annually (Konya et al., 2023); however, the actual number of patients with brucellosis may be 10–25 times the number of reported cases, largely due to misdiagnosis and underdiagnosis, particularly in endemic areas (Liu et al., 2023).

The clinical manifestations of brucellosis are primarily associated with the inflammatory response of the infected organ (Majzoobi et al., 2022) and typically include fever, headache, musculoskeletal pain, general fatigue, weight loss, and hyperhidrosis (Pappas et al., 2005). As the disease progresses, complications affecting various body systems may arise. Literature indicates that the incidence of *Brucellar* spondylitis ranges from 2 to 60% (Spernovasilis et al., 2024). This condition predominantly occurs in elderly patients and often results in the destruction of the adjacent vertebral edge bone, leading to morphological changes in the lumbar spine. Patients may experience symptoms such as low back pain, stiffness, and occasionally numbness in the lower limbs, among other discomforts. If the infection continues to spread, it may lead to the development of paravertebral abscesses, spinal epidural abscesses, and other conditions impacting the nervous system (Yin et al., 2018). Furthermore, delays in diagnosis and treatment can result in chronicity, significantly impairing the ability of patients to work and adversely affecting human health and the economic development of animal husbandry. Recent years have seen considerable advancements in the understanding of *Brucellar* spondylitis, yet no bibliometric analysis has specifically addressed this area of research. Thus, this article holds significant importance for exploring the evolution of the topic and identifying cutting-edge trends in *Brucellar* spondylitis research.

CiteSpace is a document visualization analysis software developed within the framework of scientometrics and knowledge visualization. It is specifically designed to identify the potential knowledge embedded in scientific documents. This software assists researchers in understanding the foundational knowledge of a subject, locating seminal works in the field, uncovering emerging research frontiers, and elucidating the context of research development (Chen, 2016).

VOSviewer (Visualization of Similarities viewer) is a professional software developed by Leiden University Science and Technology Research Center in the Netherlands, designed for bibliometrics and scientific visualization. The tool can effectively display the co-occurrence relationship among keywords and support several algorithms for topic clustering analysis, thereby identifying the popular keywords and core topics in the research field (Arruda et al., 2022).

This study utilized the Web of Science database to search for relevant literature and employed CiteSpace and VOSviewer software to conduct a visual analysis of *Brucellar* spondylitis. This study analyzed theoretical research on *Brucellar* spondylitis from the past 30 years, aiming to elucidate the research distribution, collaborative relationships, research hotspots, and developmental trends associated with this disorder. The findings are crucial for promoting academic development in this field, fostering interdisciplinary collaboration, and enhancing clinical practice.

## Materials and methods

### Data sources and retrieval strategies

This study utilized data sourced from the literature on *Brucellar* spondylitis within the Web of Science Core Collection (WoSCC) database. The time frame for the data collection spans from January 1, 1984, to December 31, 2023. The search strategy employed was as follows: [TS = (Brucellosis) OR TS = (“Brucella Infection”) OR TS = (“Brucella Infections”)] AND [TS = (Spondylitis) OR TS = (“Spine infection”) OR TS = (“brucellar spondylitis”) OR TS = (“spinal brucellosis”)]. The literature search was conducted on May 1, 2024.

**Figure 1:**
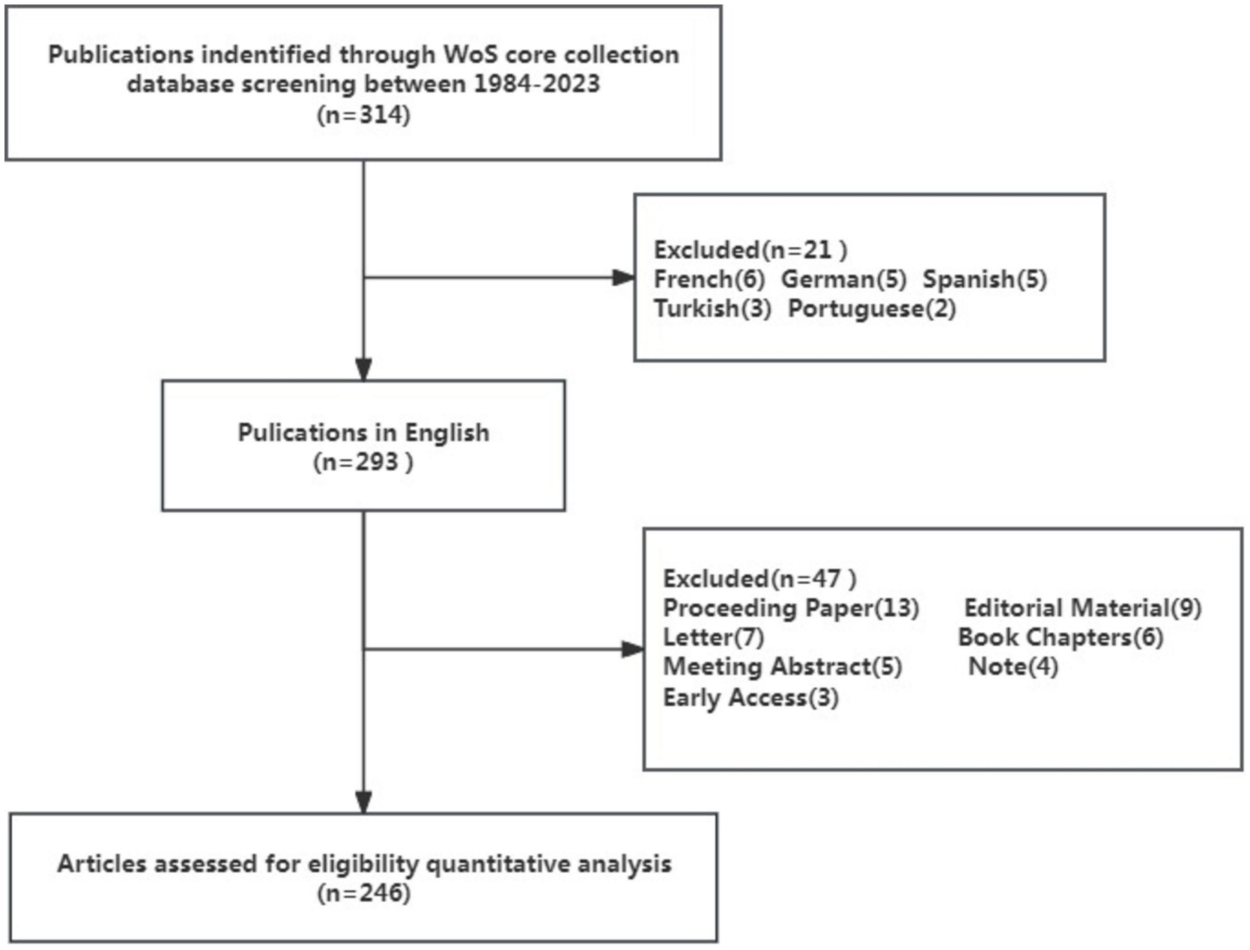
Flow diagram of included publications.

This study encompassed English-language monographs and review papers that focused on *Brucellar* spondylitis. The language setting was English, and the document types considered were articles or review articles. The document data export included a complete record and citation of references, with all records exported as plain text files. The exported record was named “download_1-246.txt” to preserve the original data. Using the review function of CiteSpace software, 0 English documents were reviewed and subsequently eliminated. Ultimately, 246 qualified documents were included in the study. The technical research route is illustrated in Figure 1.

### Analytical method

This study utilized Microsoft Excel (2019) for data management and the analysis of publication trends. CiteSpace software (version 6.1.6) was employed to conduct collaboration network analysis involving countries, institutions, and authors, as well as to perform co-occurrence analysis of keywords and co-citation analysis of references. VOSviewer software (version 1.6.20) was used for keyword co-occurrence and hotspot analysis.

The CiteSpace visual knowledge graph consists of nodes and the connections between them. Nodes represent elements such as authors, journals, countries, keywords, and other relevant entities, whereas connections indicate the intensity of cooperation among these nodes. Each group of cluster labels signifies the relevance and similarity of keywords within the cluster. In co-citation and keyword analysis, CiteSpace provides two key indicators: the module value (Q value) and the average silhouette value (S value), which are based on the network structure and the clarity of clustering. These indicators serve as a basis for evaluating the effectiveness of the visual representation. In general, the Q value falls within the interval [0, 1]; a Q value greater than 0.3 indicates a significant community structure. An S value of 0.7 suggests a high degree of clustering closeness and similarity, whereas a value above 0.5 is typically considered reasonable for clustering (Chen, 2006). Centrality is employed to assess the importance and influence of specific nodes (such as authors, documents, or keywords) within the academic network. By analyzing centrality, one can reveal the position of a specific node in academic research and its contribution to the broader research field. The time zone view (Timezone) illustrates the relationships and spans between clusters over time, effectively displaying the historical duration and interconnections of each cluster document. Additionally, emergent words highlight emerging topics within a particular research field, potentially indicating the research frontiers in that domain.

VOSviewer is also a professional visualization tool for building network graphs (Zhou et al., 2024). It can quantitatively analyze literature data and draw visual maps to reveal hot topics and trends in specific research fields. In the VOSviewer graph, nodes represent different keywords, the size of nodes indicates the frequency of occurrence, the lines between nodes indicate the correlation between keywords, and the thickness of the lines reflects the strength of the keyword connection.

## Results

### Publishing trend

**Figure 2:**
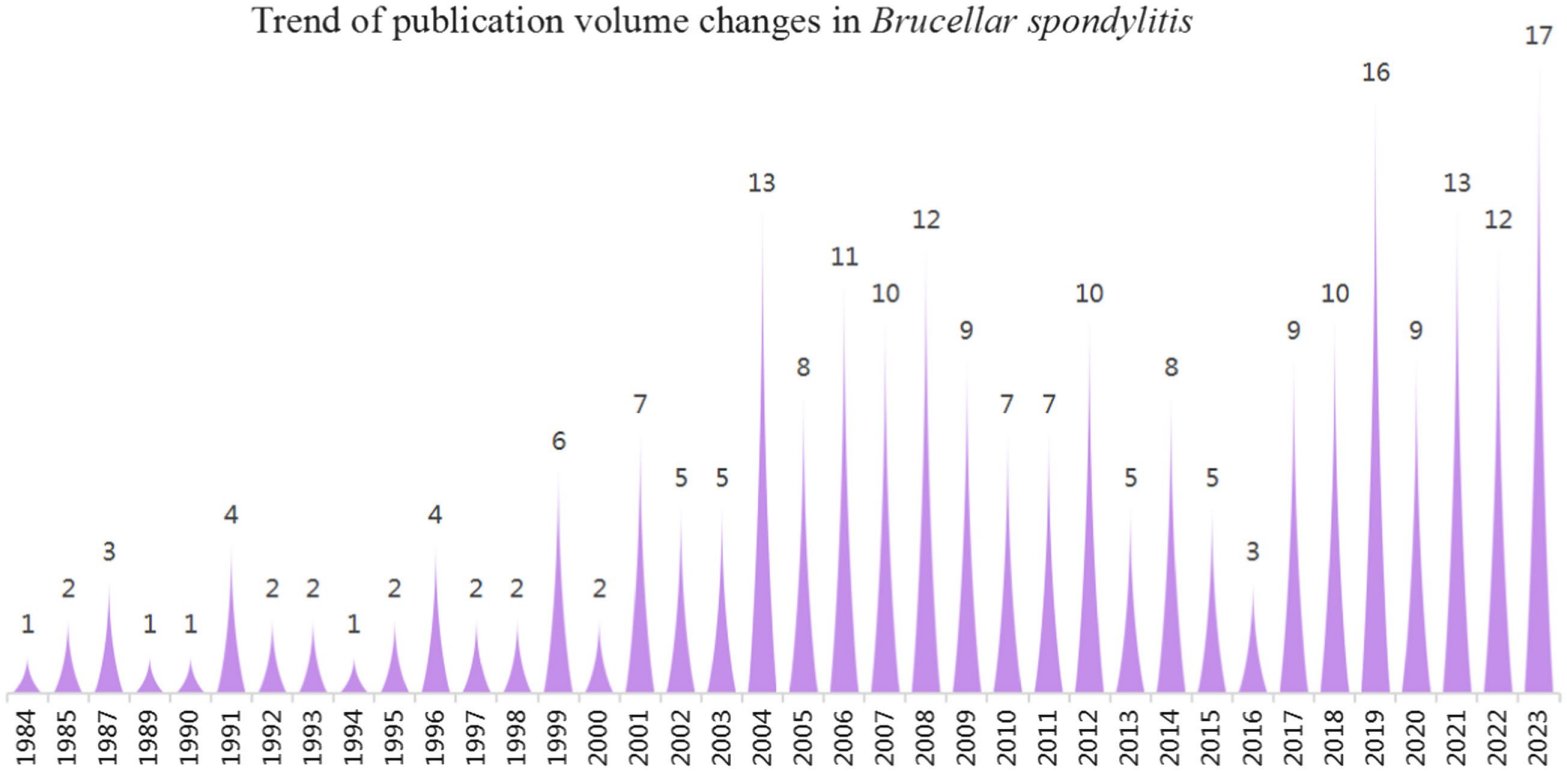
Number of articles published in the field of *Brucellar spondylitis* from 1984 to 2023.

Between 1984 and 2023, a total of 246 documents on *Brucellar* spondylitis were retrieved, comprising 221 articles and 25 review articles. The number of research publications generally showed a fluctuating upward trend. As shown in Figure 2, the number of articles published between 1984 and 2003 developed slowly, and the early stages received relatively little attention. In 2004, a notable breakthrough occurred, resulting in an annual publication of 13 papers, indicating substantial advancement of research in this field. The overall trend fluctuated over the next decade and failed to sustain the progress of 2004. Since 2017, the number of articles published showed a consistent annual increase, culminating in a record high of 17 articles in 2023. This phenomenon shows that as research on *Brucellar* spondylitis advances, scholars around the world have increasingly focused on the disease, facilitating the swift progress of related fields.

### Country/region and institution

**Figure 3:**
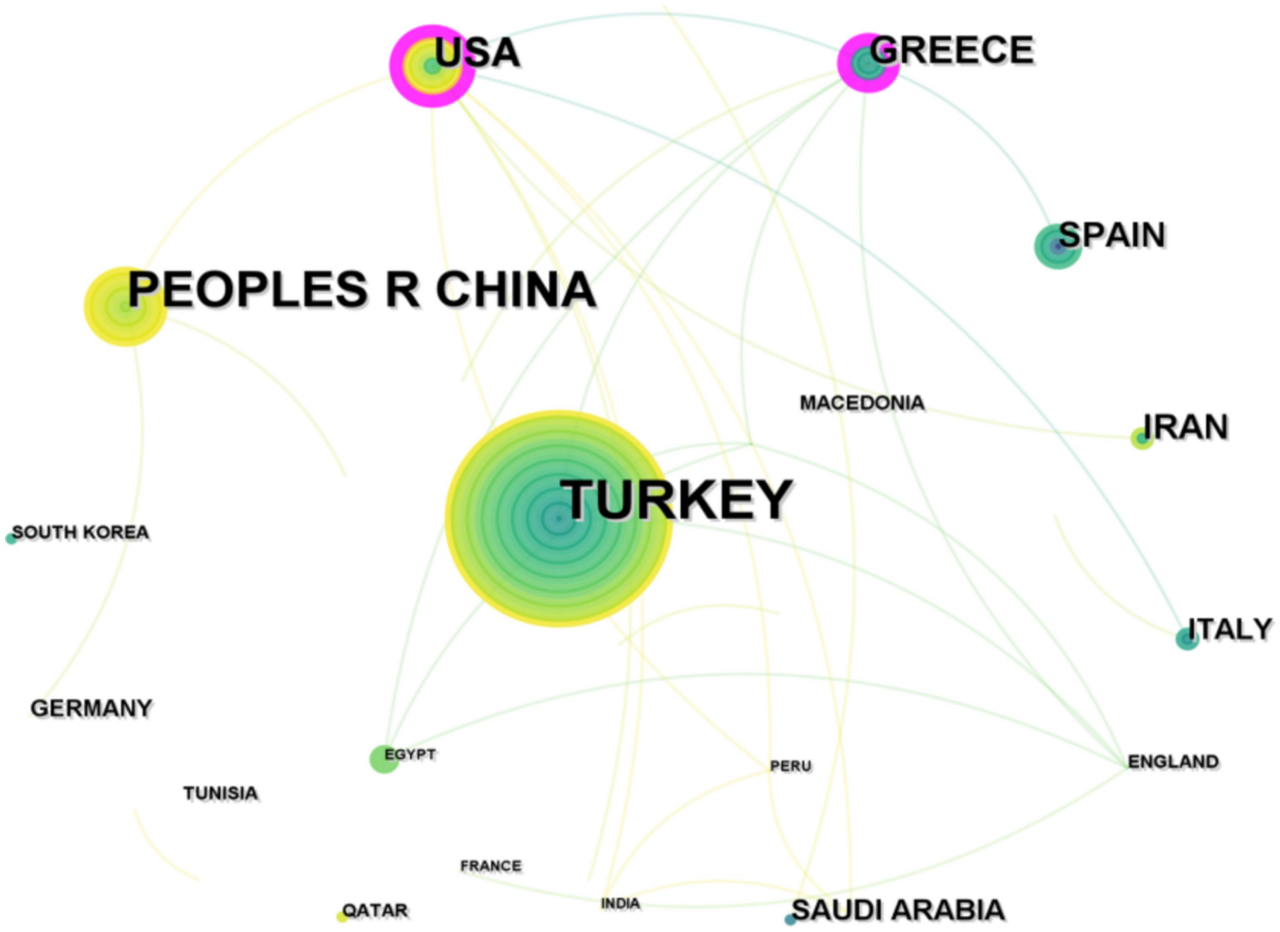
National cooperative network co-occurrence map in the field of *Brucellar spondylitis*.

Research on *Brucellar* spondylitis has yielded articles from 39 different countries/regions. As shown in Table 1, the Republic of Turkey leads with the highest number of publications, with 80 articles, accounting for 36.2% of the overall literature. The People’s Republic of China follows with 47 articles, representing 19.1% of the total. Figure 3 presents a visual network map of the partnership between countries and regions. The nodes in the network graph represent countries/regions, the size of nodes reflects the number of articles published by countries/regions, and the lines between nodes represent the cooperative relationship between countries/regions. The analysis of the national cooperation network revealed 30 cooperation records among countries, with a network density of 0.0405, indicating an existing cooperative tie between countries. The map clearly indicates strong academic collaborations among Turkey, Greece, and the UK. The country with the strongest centrality was the United States (0.34), indicating that the United States had a high influence and activity in this field.

**Table 1:** Top 10 countries/regions for related publications.

Rank	Count	Centrality	Countries/regions	Rank	Count	Centrality	Countries/regions
1	80	0	Turkey	6	13	0	Iran
2	47	0.09	People’s Republic of China	7	8	0.05	Italy
3	21	0.34	USA	8	8	0	Saudi Arabia
4	16	0.25	Greece	9	5	0	Germany
5	15	0	Spain	10	4	0	Macedonia

**Figure 4:**
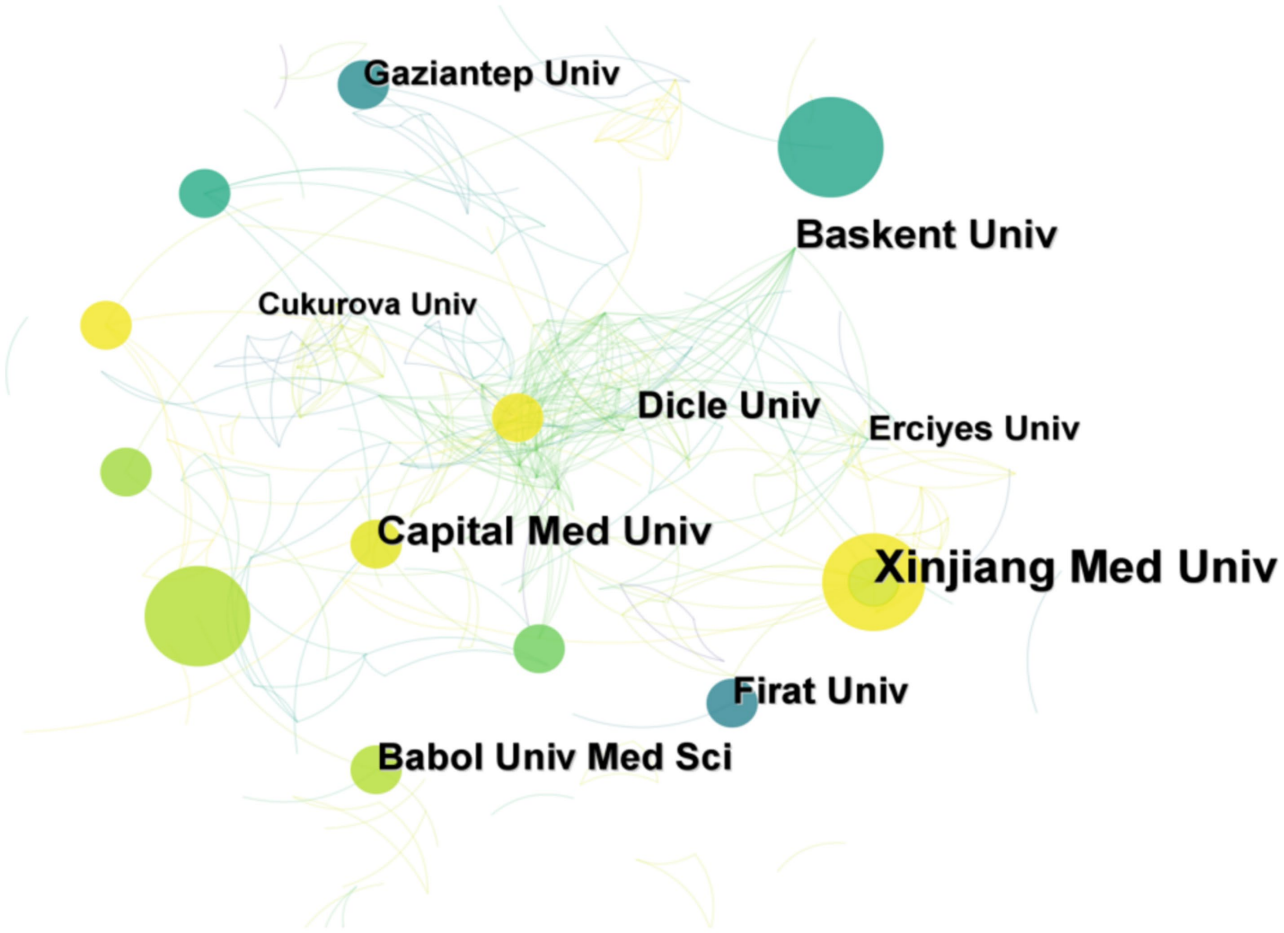
Collaborative network visualization in the field of *Brucellar spondylitis*.

The study area comprised 337 research institutions. As shown in Table 2, the research institution with the most publications was Xinjiang Med Univ (10 articles, 4.1%), and the institution with the earliest publication was Cukurova Univ (1999) in Turkey. Two of the top five most productive institutions were from the People’s Republic of China. In terms of centrality, the centrality of each institution was 0 or 0.01, indicating that the institutions were relatively isolated in the research field and did not form a broad academic exchange and cooperation network. Figure 4 illustrates the visualized network map of institutional partnerships, with 337 research institutions and 452 institutional collaborations in this research field. The network density of agency cooperation was 0.008, indicating few cooperative relationships between agencies. Xinjiang Med Univ and Baskent Univ have made great contributions in this field.

**Table 2:** Top 10 institutions for related publications.

Rank	Count	Centrality	Institutions	Rank	Count	Centrality	Institutions
1	10	0	Xinjiang Med Univ	6	6	0	Firat Univ
2	7	0	Baskent Univ	7	5	0	Gaziantep Univ
3	7	0	Capital Med Univ	8	5	0	Erciyes Univ
4	6	0	Dicle Univ	9	4	0	Cukurova Univ
5	6	0	Babol Univ Med Sci	10	3	0	Inonu Univ

### Author and co-cited author

**Figure 5:**
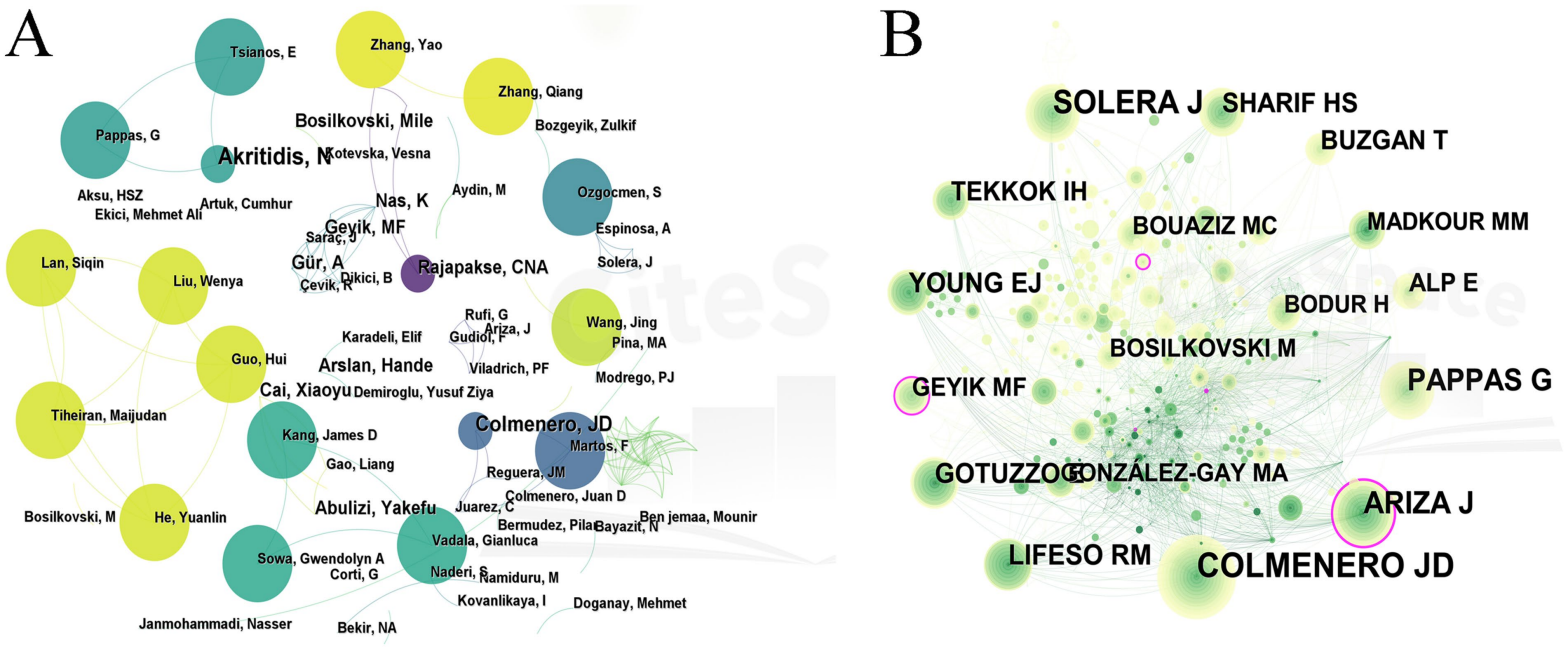
Authors analysis on *Brucellar spondylitis*. **(A)** Co-occurrence network of authors. **(B)** Co-occurrence map of cited authors.

Author collaboration network analysis can reveal research teams and major contributors in a certain field. Figure 5A shows a visual network map of author collaboration, where nodes represent authors, the size of nodes reflects the number of articles published by authors, and the lines between nodes represent the cooperative relationship between authors. A total of 681 authors published articles on *Brucellar* spondylitis in the past 30 years, with 1,241 cooperative records between the authors. The authors who contributed the most articles in this field were Colmenero, Juan D. and Solera, Javier T. (6 articles), and the earliest author of the article was Colmenero, Juan D. (1991). Thus, Professor Colmenero and Juan D have significant academic influence in the field of *Brucellar* spondylitis. The network density of authors’ collaboration was 0.0054, indicating low collaboration between authors and their teams. Table 3 shows that the centrality of all authors was 0, which indicated that the research among the authors was relatively independent, and there is a lack of extensive academic communication and cooperation networks.

**Table 3:** Top 10 authors and cited authors for related publications.

Rank	Count	Centrality	Authors	Rank	Count	Centrality	Cited authors
1	6	0	Colmenero, Juan D.	1	118	0.09	Colmenero, Juan D.
2	6	0	Solera, Javier T.	2	91	0.07	Solera, Javier T.
3	5	0	Pappas, Georgios	3	76	0.24	Javier Ariza
4	5	0	Sheng, WeiBin	4	67	0.07	Pappas, Georgios
5	4	0	Saltoglu, Nese	5	49	0.02	Lifeso, RM
6	4	0	Zhang, Qiang	6	46	0.03	Young, Erica J.
7	4	0	Nas, Kemal	7	43	0.1	Gotuzzo, Eduardo
8	4	0	Roushan, M. Reza	8	42	0.03	Sharif, Hira Salman
9	4	0	Geyik, Mehmet Faruk MF	9	39	0.06	Tekkok, Ismail Hakki
10	3	0	Rajapakse, C.	10	38	0.02	Buzgan, Turan

Author co-citation analysis (ACA) is a method employed to examine the correlations among authors within a document. A high frequency of co-citations between authors indicates a strong correlation within a specific research field (Chen et al., 2010). Figure 5B presents the author co-citation map, with the size of nodes reflecting the number of authors cited. The figure shows that the most frequently cited author was COLMENERO JD (Count: 118, Centrality: 0.09), followed by SOLERA J (Count: 91, Centrality: 0.07) and ARIZA J (Freq: 76, Centrality: 0.24), who were the co-cited authors with the highest centrality.

### Published journal and co-cited journal

**Figure 6:**
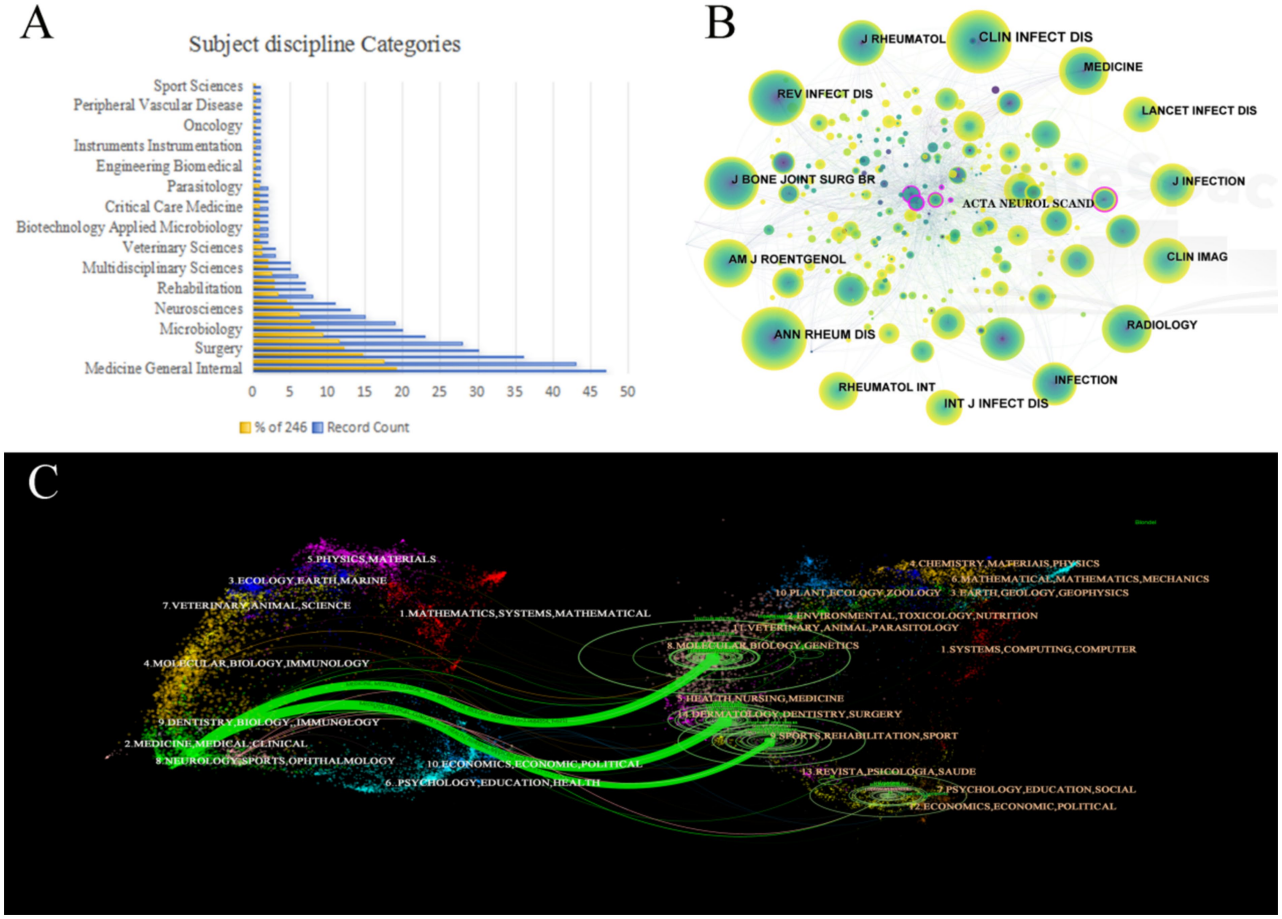
Journals analysis on *Brucellar spondylitis*. **(A)** Categories in *Brucellar spondylitis*. **(B)** Co-citation map of journals. **(C)** Superposition diagram of double journals.

The researchers have published papers across 160 journals. Table 4 shows the top 10 journals, with the JOURNAL OF INFECTION IN DEVELOPING COUNTRIES publishing the highest number of papers (6), followed by CLINICAL INFECTIOUS DISEASES, CLINICAL RHEUMATOLOGY, MEDICINE, and RHEUMATOLOGY INTERNATIONAL (5 articles). The journal impact factor (IF) reflects the average frequency with which articles from a journal are cited during a specific time (Baptista et al., 2019), and a high IF indicates that the journal’s articles are widely recognized and cited. Among the top 10 academic journals, the average IF was 2.24, and the average H-index was 82.20. The journal with the highest IF was CLINICAL INFECTIOUS DISEASES (8.2), an international peer-reviewed medical journal focusing on clinical research in the field of infectious diseases. Of the 43 categories of journals in the field Figure 6A, Medicine General Internal, Infectious Diseases, and Clinical Neurology were the major journal categories.

**Table 4:** Top 10 journals and impact factor for related publications.

Rank	Journals	Np	Nc	H-index	IF	Q
1	JOURNAL OF INFECTION IN DEVELOPING COUNTRIES	6	32	40	1.4	Q4
2	CLINICAL INFECTIOUS DISEASES	5	328	303	8.2	Q1
3	CLINICAL RHEUMATOLOGY	5	110	74	2.9	Q2
4	MEDICINE	5	476	135	1.3	Q2
5	RHEUMATOLOGY INTERNATIONAL	5	110	66	3.2	Q2
6	ACTA MEDICA MEDITERRANEA	4	5	19	0.098	Q4
7	CLINICAL IMAGING	4	83	44	1.8	Q3
8	JOURNAL OF KOREAN NEUROSURGICAL SOCIETY	4	23	29	1.4	Q3
9	NEUROSURGERY QUARTERLY	4	4	15	0	Q4
10	SPINAL CORD	4	63	97	2.1	Q3

Np, the number of publications; Nc, the number of citations without self-citations. IF, impact factor; Q, JCR quartile ranking.

Figure 6B presents the co-citation network map of journals, with node size reflecting the number of journal citations. The map shows a total of 537 co-cited journals. The most cited journal was LIN INFECT DIS (Count: 130, Centrality: 0.02, Year: 1994), followed by ANN RHEUM DIS (Count: 88, Centrality: 0.05, Year: 1987). ACTA NEUROL SCAND (Freq: 29, Centrality: 0.20) was one of the most important journals in the field of neurology.

In the study of superimposed bi-journal maps of *Brucellar* spondylitis (Figure 6C), each data point on the map represented a scientific journal. The map is divided into two sections: the journals that are referenced on the left and the cited journals on the right. This method shows key information such as the distribution of subject papers, citation trajectory, and center of gravity shift (Chen and Leydesdorff, 2014). Among them, there are three outward citation paths in the fields of medicine, biology, neurology, and sports medicine on the left side of the figure, highlighting their importance in citations. At the same time, molecular biology, genetics, sports medicine, and rehabilitation were the main sources, and molecular biology and genetics had the highest citation rate (*Z* value = 3.968).

### References and co-cited references

**Figure 7:**
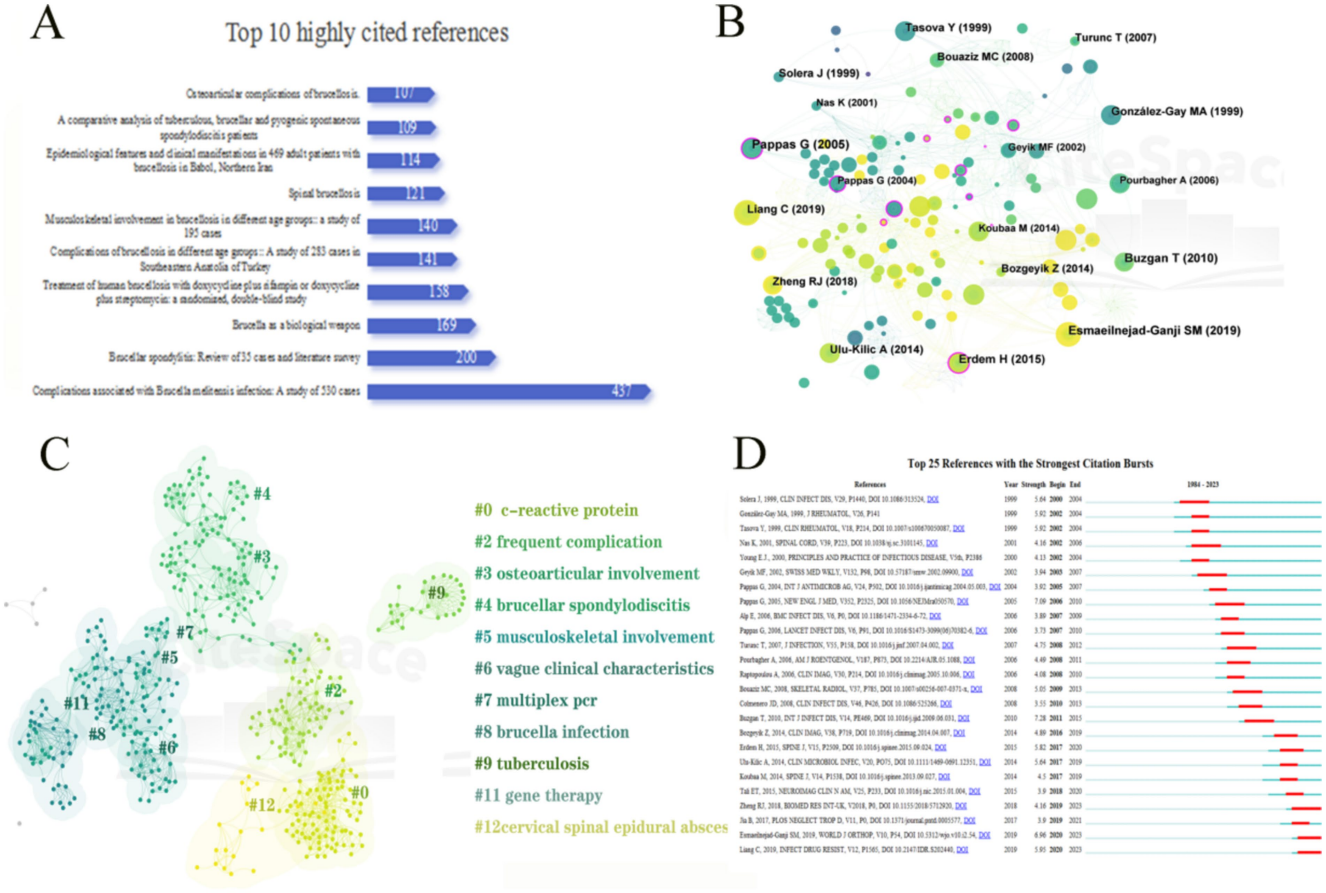
References analysis on *Brucellar spondylitis*. **(A)** Top 10 highly cited references on *Brucellar* spondylitis-related research. **(B)** Co-citation reference network map in the field of *Brucellar spondylitis*. **(C)** Cluster map of co-cited references in the field of *Brucellar spondylitis*. **(D)** Burst terms in co-cited references in the field of *Brucellar spondylitis*.

Of the 246 included articles in this field, Figure 7A shows the top 10 cited articles, comprising a significant number of retrospective case series studies from different regions (6), 2 review articles, 1 double-blind randomized study comparing the efficacy of different antibiotic treatments, and 1 double-blind randomized study comparing the efficacy of different antibiotic treatments. One article expressed the possibility of *Brucella* as a biological weapon.

The co-cited reference map reveals widely cited classical literature and foundational work in the research field, which often have had a profound impact on subsequent research. Figure 7B shows the network map of co-cited references, where nodes represent co-cited references, and the size of nodes indicates the citation frequency of the literature. Among the 787 co-cited references, 9 papers were cited more than 10 times, and Table 5 lists the top 10 co-cited references. Among the most frequently cited articles, articles in the International Journal of Antimicrobial Agents ranked first, with 100 citations (Title: New approaches to the antibiotic treatment of brucellosis). This article revealed that tigecycline may help achieve monotherapy or shorten the treatment time, or changing the acidic intracellular environment inhabited by *Brucella* may improve the efficacy of antibiotic treatment (Pappas et al., 2005). The second most cited article was published in 2010 in the International Journal of Infectious Diseases, with 887 citations (Title: Clinical manifestations and complications in 1028 cases of brucellosis: a retrospective evaluation and review of the literature). The study by the team of Buzgan T. used a variety of laboratory tests to confirm the diagnosis of brucellosis, retrospectively analyzed the medical records of 1,028 patients, and compared them with other literature, showing that the disease can cause serious diseases, such as osteoarthritis, central nervous system disease, and endocarditis. He also expressed the serious circumstances associated with the disease in Turkey and suggested that the most appropriate treatment method and duration of treatment are not yet available (Buzgan et al., 2010). The third ranked article (title: Osteoarticular manifestations of human brucellosis: a review) was published in the World Journal of Orthopedics and was cited 164 times. Esmaeilnejha-ganji S M et al. gave a detailed description of the different types of *Brucella* osteoarthritis and emphasized the importance of early diagnosis and treatment. The articles that ranked 4th to 9th were all retrospective studies of case series, which reported the epidemic status and clinical characteristics of the disease in various countries or regions (Erdem et al., 2015; González-Gay et al., 1999; Liang et al., 2019; Solera et al., 1999; Taşova et al., 1999; Ulu-Kilic et al., 2014). The 10th ranked article reviewed the clinical, biological, and imaging features of *Brucellar* spondylitis (Chelli Bouaziz et al., 2008). Among the 787 co-cited references, Professor Gorgulu A’s study, which had the highest centrality ranking, provided important information on spinal epidural abscess caused by *Brucella* infection (Görgülü et al., 2006), and this article was cited 42 times.

**Table 5:** Top 10 references on *Brucellar* spondylitis-related research.

Rank	Total citations	Year	Cited reference
1	100	2005	New approaches to the antibiotic treatment of brucellosis
2	887	2010	Clinical manifestations and complications in 1028 cases of brucellosis: a retrospective evaluation and review of the literature
3	164	2019	Osteoarticular manifestations of human brucellosis: a review
4	66	2015	Comparison of brucellar and tuberculous spondylodiscitis patients: results of the multicenter
5	137	1999	Osteoarticular complications of brucellosis in an Atlantic area of Spain
6	45	2019	Spinal brucellosis in Hulunbuir, China, 2011–2016
7	425	1999	Brucellar spondylitis: review of 35 cases and literature survey
8	174	1999	Osteoarticular involvement of brucellosis in Turkey
9	155	2014	Update on treatment options for spinal brucellosis
10	154	2008	Spinal brucellosis: a review

Figure 7C depicts the cluster map of co-cited references, where modularity *Q* = 0.8677 and weighted mean silhouette *S* = 0.9394. Modularity *Q* > 0.3 and silhouette *S* > 0.7 indicated that the closeness and similarity of the reference keywords in this study were high, and the clustering effect was good. In the atlas, the depth of the color represents the formation time of the cluster of the study cluster; a dark color means that the cluster formed early, whereas a light color indicated that it was close to the current stage of the study. Therefore, the development of this field could be roughly divided into three research stages. In the early research period (1995–2005), there were 5 instances of musculoskeletal involvement, 6 instances of nonspecific clinical features, 7 instances of multiplex PCR, 8 instances of *Brucella* infection, and 11 instances of gene therapy. In the subsequent study (2005–2015), 3 cases of osteoarticular involvement, 4 cases of *Brucellar* spondylitis, and 9 cases of tuberculosis were found. Currently (from 2015 to now), the research contents were as follows: 0 C-reactive protein, 2 common complications, and 12 cervical spinal epidural abscess.

Citation burst analysis is used to reveal the highly influential and important literature in a specific time period by identifying the studies with a surge in the number of citations during that period. On the basis of the strongest citation burst (bursts), Figure 7D shows that the first citation burst began in 2000, namely, Solera J, et al. After assessing the clinical presentation and treatment outcome of 35 cases of *Brucellar* spondylitis, no deaths or serious complications were observed. Nevertheless, patients suffer from considerable pain and may miss work due to their illness (Solera et al., 1999). The citation emergence intensity of the top 25 references ranged from 3.55 to 7.28. Among them was Buzgan T, et al. Through the analysis of 1,028 cases and a comprehensive evaluation combined with the existing literature, the study revealed that *Brucellar* spondylitis may lead to a serious pathological state and remains a difficult public health problem in Turkey. We should enhance our understanding of the diverse clinical features of the disease and its complications to optimize diagnostic and treatment strategies (Buzgan et al., 2010).

### Keyword analysis

**Figure 8:**
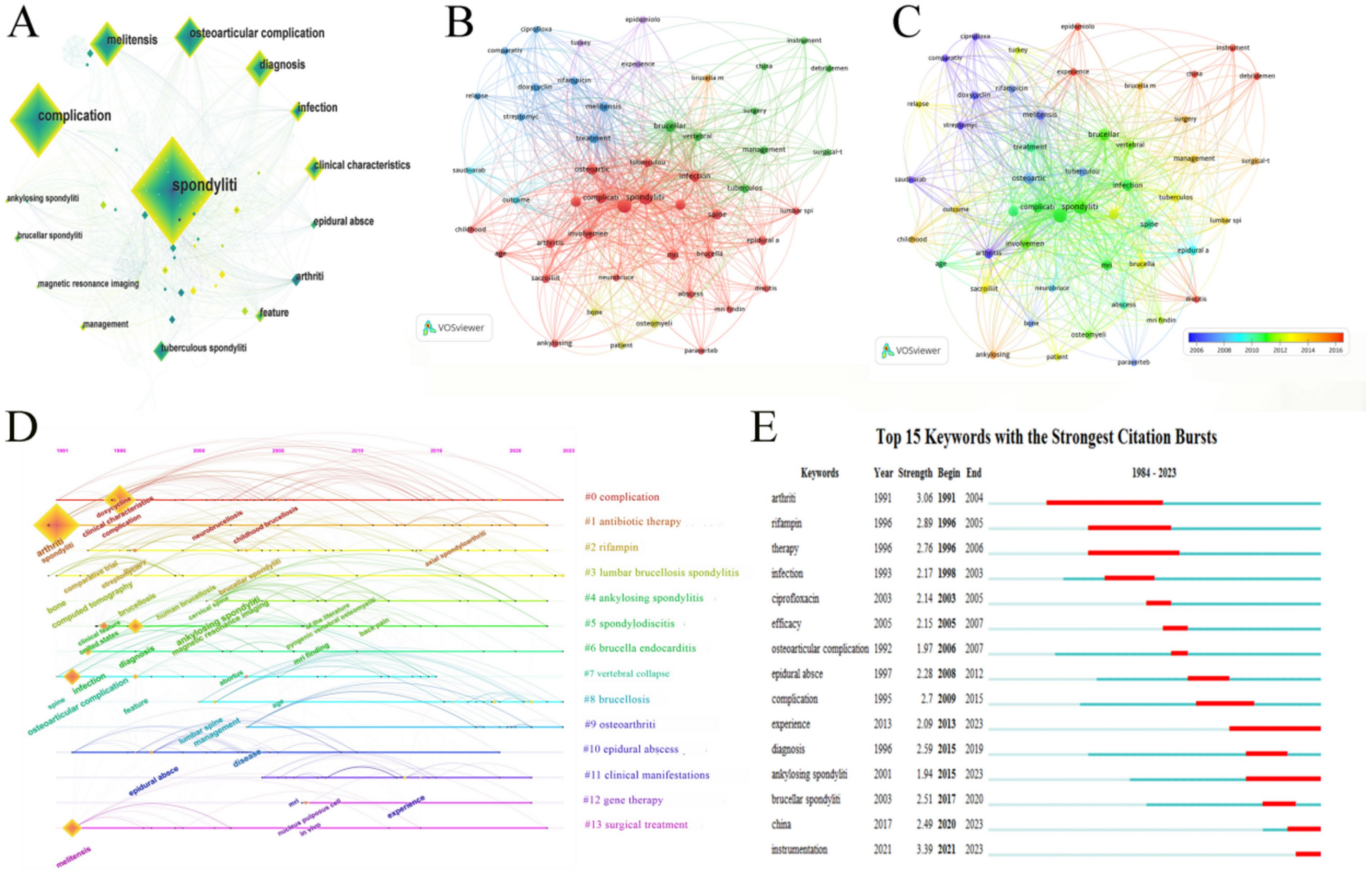
Keyword analysis on *Brucellar spondylitis*. **(A)** Network map of the co-occurrence keywords. **(B)** Network visualization map of keywords based on VOSviewer. **(C)** Overlay visualization map of keywords based on VOSviewer. **(D)** *Brucellar spondylitis* Keyword Timeline Clustering Map. **(E)** Burst terms in keywords in the field of *Brucellar spondylitis*.

Keyword co-occurrence analysis can help us understand the knowledge structure of the subject domain and explore the trends (Zhang et al., 2022). Keyword frequency analysis and statistics of the occurrence frequency of specific keywords in the literature can reveal the important research directions and clinical concerns in the research of *Brucellar* spondylitis. As shown in Table 6 and Figure 8A, 404 keywords appeared in this field, with 9 keywords appearing more than 20 times, and “spondylitis” (146) ranked at the top. “Arthritis” was the most central keyword (0.33), appearing 19 times. This was followed by “ankylosing spondylitis” (0.27), “infection” (0.24), and “management” (0.21).

**Table 6:** Top 10 keywords in the field of *Brucellar* spondylitis.

Rank	Count	Centrality	Year	Keyword
1	146	0.1	1991	Spondylitis
2	66	0.08	1995	Complication
3	35	0.09	1992	Melitensis
4	35	0.2	1992	Osteoarticular complication
5	30	0.14	1996	Diagnosis
6	27	0.24	1993	Infection
7	21	0.06	1995	Clinical characteristics
8	20	0.09	1997	Epidural abscess
9	19	0.33	1991	Arthritis
10	18	0.13	1996	Feature

Clustering keywords can provide a comprehensive and accurate understanding of high-frequency keywords identify the correlation between different research topics and directions and reveal the internal structure and research hotspots of a particular field. On the basis of keyword co-occurrence analysis keyword cluster analysis was carried out. Figure 8D shows the timeline visualization map of keyword cluster analysis. The keywords were arranged according to the timeline to show the evolution trend of different keyword groups: #1 (antibiotic therapy) #2 (rifampin) #3 (lumbar brucellosis spondylitis) #4 (ankylosing spondylitis) #5 (spondylodiscitis) #6 (*Brucella* endocarditis) #7 (vertebral collapse) #8 (brucellosis) #9 (osteoarthritis) #10 (epidural abscess) #11 (clinical manifestation) #12 (gene therapy) and #13 (surgical treatment). The Cluster ID is the number after keyword clustering and the number is shown in the figure as #0 #1 etc. The larger the scale of the cluster the larger the number of members included in the cluster; the smaller the scale of the cluster the smaller the number of members. In Figure 8B the red clusters were mainly composed of “spondylitis,” “complication,” and “infection.” This cluster mainly involved various complications caused by *Brucellar* spondylitis infection. The blue clusters were mainly composed of “treatment,” “rifampicin,” “doxycyclin,” and “relapse.” This cluster focused on the treatment and prognosis of this disease. The clusters in green were mainly composed of “vertebral,” “brucellar,” “surgery,” and “management.” This cluster addressed the surgical treatment and management of spondylitis caused by *Brucella* infection. Figure 8C shows the evolution trend of hot topics in the field of *Brucellar* spondylitis. From 2006 to 2010 researchers paid attention to the selection of various antibacterial agents for this disease. From 2010 to 2014 researchers mainly focused on the manifestations of local tissue or organ involvement caused by this disease and the corresponding examination methods. Since 2014 the research focused on the surgical treatment and management of the disease and the sharing of related experiences.

Further keyword highlight analysis was carried out, which can effectively show the dynamics of keywords and their sudden changes in the research field, help researchers identify research hotspots and academic trends (Long et al., 2023), and provide reference for future scientific research work. Figure 8E presents a highlight map of keywords, showing the intensity of the top 15 keywords in *Brucellar* spondylitis research. The first outbreak began in 1991, with the intensity ranging from 1.94 to 3.39, and “instrumentation” (3.39) had the highest intensity. The length of the red line represents the duration of prominence, and “arthritis” was the keyword with the longest duration of prominence from 1991 to 2014. The key words that have emerged so far include “experience,” “ankylosing spondylitis,” “China,” and “instrumentation.”

## Discussion

### General information

In recent years, advancements in the study of *Brucellar* spondylitis have led researchers to achieve substantial progress in its etiology, pathogenesis, diagnosis, and treatment. Despite the annual growth of research in this field, the systematic summary and analysis of research hotspots and development trends are still insufficient. This study used bibliometric analysis to focus on the research field of *Brucellar* spondylitis, offering a comprehensive overview of *Brucellar* spondylitis and providing an important reference for future research direction, especially for strengthening international cooperation, expanding research fields, and improving research depth.

The publication trend of *Brucellar* spondylitis-related literature clearly illustrates the dynamic evolution of research in this field. Although the causative agent of brucellosis was identified as early as the end of the 18th century (Roop et al., 2021), research on *Brucellar* spondylitis progressed relatively slowly over the following century, resulting in the disease being often misdiagnosed as other diseases such as ankylosing spondylitis or lumbar disc herniation in its early stages. This may be related to the lack of a unified name of the disease in the early stage, which was once called “Crimean fever,” “Maltese fever,” “Mediterranean fever,” and “wavy fever” (Moreno et al., 2022). Until 1920, Alice Evans, a famous American microbiologist, proposed the name “brucellosis” in memory of David Bruce, and this term has been used ever since (Meyer and Shaw, 1920). The number of relevant publications from 1984 to 2003 was relatively small. Since 2004, significant progress has been made in the research of *Brucellar* spondylitis, evidenced by a marked growth in published articles. This may be attributed to advancements in diagnostic techniques, enhancements in scientific research methods, and the increasing attention to zoonotic diseases in the global health field. Since 2019, the number of articles has shown an explosive growth, indicating that brucellosis remains a global public health challenge, attracting increased interest from researchers and clinicians.

The publications of various countries and research institutions objectively reflect the scientific research level and influence of related research fields, as well as the collaborative research results among countries. The Republic of Turkey holds a leading position for the number of publications and international influence, having made outstanding contributions to the research of *Brucellar* spondylitis. The Republic of Turkey, despite the publication of 80 articles, had a centrality value of 0. This value may be due to the failure of the country’s research output to form a strong cooperation or influence relationship in the network, which may be related to the low cooperation density in other countries. Centrality values reflect the position and influence of the country in the cooperative network, rather than only the number of articles published. Turunc T et al. compared the characteristics of tuberculous spinal discitis (TS group), *Brucellar* spinal discitis (BS group), and suppurative spinal discitis (PS group) in a prospective study and found a significantly higher rate of microbial detection and high fever in the BS group. By contrast, the TS group exhibited significantly higher rates of chronic renal failure, systemic symptoms, psoas abscess, history of tuberculosis, high blood sedimentation rate, posterior column involvement, and/or surgical treatment (Turunc et al., 2007). In recent years, China has demonstrated strong growth in research output in this field, and the top five productive institutions included Xinjiang Medical University and Capital Medical University. The prevalence of brucellosis in humans may be an important factor contributing to the high number of publications in China. In addition, the frequency of publication may be influenced by the country’s academic and public health resources, rather than simply reflecting research expenditures. In areas where brucellosis is endemic, patients with unexplained fever, joint pain, and lower back pain should be considered for local computed tomography (CT) or magnetic resonance imaging (MRI) to rule out the possibility of *Brucellar* spondylitis (Garfield, 2006). In the future, the rapid spread and application of new technologies and methods must be promoted to enhance the global understanding and prevention of *Brucellar* spondylitis.

Journal IF is an important indicator to measure the academic influence of journals in this research field. It is calculated by comparing the average number of citations received by each article in the first 2 years (Garfield, 2006). We generally believe that the IF of a journal is closely related to the impact of articles from that journal (Callaham et al., 2002). H-index is an index to measure the academic influence of scholars, which was proposed by Jorge Hirsch, an American physicist, in 2005 (Hirsch, 2005). It represents the number and level of academic output of scholars. CLINICAL INFECTIOUS DISEASES had the highest impact factor (8.2) and H index (303) among the top ten journals with the largest number of articles, signifying its remarkable academic influence and citation rate in the field of infectious diseases and establishing it as an authoritative journal for related research. The journal with the largest number of articles was the JOURNAL OF INFECTION IN DEVELOPING COUNTRIES, with an impact factor and H-index of 1.4 and 40, respectively. Despite its relatively low academic influence, as a journal focusing on the study of infectious diseases in developing countries, it has become an important platform for showcasing research results in the region. Other active professional category journals and multidisciplinary journals, such as CLINICAL RHEUMATOLOGY, CLINICAL IMAGING, and MEDICINE, also publish high-quality studies on *Brucellar* spondylitis in the fields of rheumatology, radiology, and general medicine, respectively. The topic has attracted wide attention from researchers in different disciplines. According to the statistics of journal categories, the field of *Brucellar* spondylitis mainly involves the research topics of internal medicine, infectious disease, clinical neurology, rheumatology, medical imaging, and pharmacology, establishing it as a multidisciplinary research hotspot.

Author analysis is used to understand the core contributors in a particular research field and reveal the collaborative relationships between scholars and the groups of authors who have made important contributions to the development of the field. Colmenero, Juan D. and Pappas, G are highly influential scholars in the field of *Brucellar* spondylitis, and their research results have significantly promoted the diagnosis, prevention, and treatment of this disease. Colmenero, Juan D., the most published and cited author in the field of *Brucellar* spondylitis, reported an important prospective study in 1996 that systematically summarized the clinical presentation, complications, and their diagnosis and treatment of 530 patients with *Brucella* infection (Colmenero et al., 1996). Their study not only improved the understanding of the clinical manifestations and complications of brucellosis but also provided a scientific basis for its early diagnosis and treatment, which has a far-reaching impact on the prevention and control of brucellosis in the world. Pappas, G, also an authoritative scholar in the field of brucellosis, elaborated the possibility of *Brucella* as a biological weapon and its related characteristics, and they offered a comprehensive assessment of the historical and realistic nature of its potential threat (Pappas et al., 2006). In addition, they provided a strong basis for international public health interventions through the epidemiological studies of brucellosis worldwide (Pappas et al., 2006). The research results of the two scholars have yielded breakthroughs in the early diagnosis and treatment of *Brucellar* spondylitis. These contributions have not only been widely recognized in the academic community but also play an important role in the global public health prevention and control framework.

The paper “Complications associated with *Brucella melitensis* infection: A study of 530 cases” ranked first in the top 10 citations. In this prospective clinical study, Colmenero et al. (1996) introduced the epidemiology, clinical manifestations, etiological and immunological examination results, and treatment effects of brucellosis, as well as analyzed the relationship between different factors and the severity of the disease. ESR > 40 mm/h and α2-globulin >7.5 g/L may lead to complications of this disease. The 10th article was also from Colmenero, JD, a professor, who used prospective studies to elucidate the clinical manifestations, imaging characteristics of *Brucella* osteoarticular complications, and effects of medical treatment and surgical treatment. Their results suggested that patients with paravertebral abscess, spinal cord compression, or destructive spondylitis resulting in severe and persistent pain require a combination of medication and surgery to improve recovery. Solera, J, Gur, A, Geyik, MF, Roushan, MRH, and Turunc, Tuba also analyzed the clinical characteristics and complications of the disease in various regions (Solera et al., 1999; Turunc et al., 2007; Gür et al., 2003; Mehmet et al., 2002; Roushan et al., 2004). Solera, J et al. analyzed 35 cases of *Brucellar* spondylitis and identified back pain, neck pain, fever, and general malaise as the main clinical manifestations of the disease. Gur, A and Geyik, MF both reported osteoarthritis as the most common complication of brucellosis in the Turkish region, accounting for 69% of the total number of cases. Gur, A’s study reported a treatment failure rate of 5%. Geyik, MF pointed out that *Brucella* infection can affect musculoskeletal disorders in people of different ages, and female patients are more likely to be affected than their male counterparts. The results of Roushan, MRH’s study indicate that most patients with this disease come from rural areas, and spring or summer is the predominant period of onset. Major risk factors included fresh cheese, animal husbandry, laboratory workers, and veterinary occupations. Complications were more common in male patients than in female patients, and arthritis was the most common complication. The study by Turunc, Tuba et al. compared the clinical, laboratory, and imaging features of tuberculous spondylitis, *Brucellar* spondylitis, and suppurative spondylitis. Their results showed that the *Brucellar* spondylitis group had a higher microbial detection rate and higher incidence of high fever than the two other groups, whereas the tuberculous spondylitis group had a higher incidence of chronic renal failure, psoas abscess, high ESR, and/or surgical treatment than the two other groups. The article published by Pappas et al. (2006) ranked third, in which they proposed the potential threat of *Brucella* in biological warfare by introducing its characteristics. ARIZA J et al. (Pappas et al., 2005) ranked fourth, in which they compared the efficacy of using a combination of doxycycline and rifampicin with that of doxycycline and streptomycin in patients with brucellosis through a randomized double-blind principle. Their results showed that the clinical cure rate and recurrence rate of the former group were lower than those of the latter group, and the incidence of adverse reactions was slightly higher than that of the latter. However, the difference between the two groups was not significant. LIFESO, RM (Colmenero et al., 2010) reviewed the clinical, biological, and imaging features of brucellosis. The above results provide guidance for our subsequent research.

In the cluster analysis of co-cited references, the development of this field can be roughly divided into three research stages (1995–2005, 2005–2015, and 2015–present). In the early stage of the study (#5, #6, #7, #8, and #11), a series of nonspecific clinical manifestations caused by *Brucella* infection involving the whole body skeletal muscle system was initially described, and the advantages of multiplex PCR technology compared with traditional blood culture in the diagnosis of the disease and antimicrobial therapy and gene therapy strategies for the disease were proposed (Pappas et al., 2004; Pappas et al., 2005; Colmenero et al., 2010; Demirci, 2003; Vadalà et al., 2007). In subsequent studies (#3, #4, and #9), the disease was further determined as a zoonotic disease prevention and treatment measure, and the clinical characteristics, imaging manifestations, and antimicrobial treatment options of *Brucella* osteoarthritis were systematically described (Lynch, 2014; Oztekin et al., 2010; Metz et al., 2012; Menon and Sorour, 2016), which further refined and expanded the research content of the disease. In recent years (#0, #2, and #12), the pathogenesis of *Brucellar* spondylitis and treatment strategies for common complications such as osteoarticular system and nervous system caused by *Brucella* infection were mainly studied (Zhang et al., 2022; Esmaeilnejad-Ganji and Esmaeilnejad-Ganji, 2019; Papadakis et al., 2023).

### Current research status and development trends

Keyword analysis can reveal the key topics, hotspots, and trends within a study domain, helping researchers understand the knowledge structure and future development direction of the field. Through the analysis of high-frequency keywords in the field of *Brucellar* spondylitis research, the main directions and clinical concerns of current research could be revealed.

Through the co-occurrence analysis of keywords, such as “complication,” “osteoarticular complication,” and “infection,” the high frequency of “epidural abscess” and “arthritis” reflects the widespread concern about complications related to *Brucella* infection in this field (Sanaei Dashti and Karimi, 2013; Karaşahin et al., 2024). A primary focus of research in this field is the various manifestations of osteoarticular complications caused by *Brucella* infection. Post-infection complications often aggravate the patient’s condition and increase the complexity of treatment. The incidence of post-infection complications is increasing in China, especially in pastoral areas and areas with weak agricultural infrastructure (Jiang et al., 2019). This infection can affect any part of the spine and usually manifests as spondylitis, osteomyelitis, sacroiliitis, peripheral arthritis, bursitis, and tenosynovitis (Dashti and Karimi, 2013), resulting in severe pain and limited mobility. In a survey by Bozgeyik et al. (2014), the lumbar spine (60%), sacrum (19%), and cervical spine (12%) were the most common sites of involvement. Esmaeilnejad-Ganji and Esmaeilnejad-Ganji (2019) identified spondylitis as a potentially dangerous complication because it is associated with epidural, paravertebral, and psoas muscle abscesses and may cause nerve compression. Epidural abscess is a rare complication of spondylitis and may lead to permanent neurological deficits. The rapid progression of the disease is often due to delays in diagnosis and treatment (Bozbaş et al., 2016). Studies have shown (Arkun & Mete, 2011) that sacroiliitis can appear in nearly 80% of patients with focal complications, which is common in adult patients and can be manifested as low back pain. Unilateral and bilateral *Brucella* sacroiliitis have been reported (Gheita et al., 2015; Ozturk et al., 2015). Current studies suggest that high-resolution MRI is the best method for the diagnosis of spondylitis, sacroiliitis, epidural abscess, and spinal cord compression associated with brucellosis due to its high sensitivity (Bozgeyik et al., 2014; Bilgeturk et al., 2017).

Through the cluster analysis of the cited references and research topics, we found that “clinical characteristics” and “therapy” were important research topics in the field of *Brucellar* spondylitis. *Brucella* infection can lead to systemic and multi-system complications, and its clinical manifestations depend on the site of bacterial infection and its severity. As a result of its non-specific clinical symptoms, it is often confused with other spinal diseases, such as ankylosing spondylitis, which often leads to delays in diagnosis and treatment. Therefore, researchers should focus on improving the accuracy of early diagnosis to reduce misdiagnosis and missed diagnosis (Tao et al., 2020). The current treatment mainly focuses on antimicrobial therapy and surgical therapy. “Drugs commonly used in the treatment of *Brucellar* spondylitis include rifampicin, doxycycline, streptomycin, trimethoprim/sulfamethoxazole, ciprofloxacin, and gentamicin (Corbel et al., 2014).” A previous study demonstrated (Oner et al., 2012) that treatment with a triple regimen of rifampin (15 mg/kg·day), doxycycline (100 mg twice daily), and streptomycin (1 g/day) for more than 6 months can achieve complete cure of the disease. Surgical treatment has been increasingly used as a complementary treatment in *Brucellar* spondylitis (Roushan et al., 2019). Surgical intervention is required for patients with spinal abscess, vertebral collapse, bone destruction, spinal cord compression, or progressive neurological deficits who fail to respond to antibiotic treatment (Bao et al., 2017; Reşorlu et al., 2016).

Through the analysis of the emergence of keywords, “instrumentation” was the largest highlight intensity of the key words, indicating that in recent years, equipment-related research has been widely concerned and valued. Accurate diagnosis and treatment cannot be separated from the support of advanced medical equipment and technology. For example, MRI can clearly show bone and joint lesions, PCR and other examination equipment can accurately detect *Brucella* infection, and the improvement of related surgical instruments makes the treatment more accurate. “Experience” was the longest highlighted keyword, indicating that in the research process of *Brucellar* spondylitis, “experience” has always been an important research theme. The clinical symptoms of the disease are atypical and easy to be confused with other diseases (such as ankylosing spondylitis and tuberculous spondylitis). Through continuous summary and reflection in long-term clinical practice, doctors can thoroughly understand the nature and law of diseases to improve the accuracy of diagnosis and the effectiveness of treatment. In clinical practice, doctors use cutting-edge medical equipment combined with personal clinical experience to tailor personalized treatment plans for patients to improve the cure rate of *Brucellar* spondylitis and improve the quality of life of patients. In the future, intelligent diagnostic devices and surgical instruments can be further developed to automatically adjust detection parameters and therapeutic targets according to the doctor’s experience and the patient’s condition.

In general, current research in the field of *Brucellar* spondylitis is focused on the clinical features of the disease, management of osteoarticular complications, and antimicrobial and surgical treatment methods. Future research may focus on the improvement of early diagnosis methods (such as further exploration of molecular biology detection methods and serum biomarkers for rapid and accurate identification of pathogenic characteristics) and the innovation of surgical instruments.

## Limitations

The limitations of this study were as follows: (1) Limitations of data sources: The CiteSpace analysis conducted in this study relies solely on the Web of Science Core Collection database, which may result in omissions in data collection. Different databases exhibit varying levels of data coverage and update frequency, potentially affecting the comprehensiveness and accuracy of the analysis results. (2) Subjectivity of keyword selection: CiteSpace analysis is contingent upon the selection of keywords, which can vary among researchers. This subjectivity in keyword screening may influence the final analysis outcomes, potentially leading to the oversight of some significant research areas.

## Conclusion

This study employed bibliometric methods to provide a comprehensive analysis of the current research status and trends in the field of *Brucellar* spondylitis. The research hotspots in this field include the clinical characteristics of the disease, the management of complications, and treatment strategies. Future research is anticipated to concentrate on enhancing early diagnostic technologies, optimizing multidisciplinary treatment options, innovating surgical intervention methods, and formulating global prevention and control strategies.

## Data Availability

The original contributions presented in the study are included in the article/supplementary material, further inquiries can be directed to the corresponding author.
